# Prefrontal Gray Matter and Motivation for Treatment in Cocaine-Dependent Individuals with and without Personality Disorders

**DOI:** 10.3389/fpsyt.2014.00052

**Published:** 2014-05-20

**Authors:** Laura Moreno-López, Natalia Albein-Urios, José Miguel Martinez-Gonzalez, Carles Soriano-Mas, Antonio Verdejo-García

**Affiliations:** ^1^Department of Personality, Evaluation and Psychological Treatment, University of Granada, Granada, Spain; ^2^Red de Trastornos Adictivos, University of Granada, Granada, Spain; ^3^Centro Provincial de Drogodependencias, Diputación de Granada, Granada, Spain; ^4^Department of Psychiatry, Bellvitge University Hospital-IDIBELL, Barcelona, Spain; ^5^Carlos III Health Institute, Centro de Investigación Biomédica en Red de Salud Mental (CIBERSAM), Madrid, Spain; ^6^Institute of Neurosciences Federico Olóriz, University of Granada, Granada, Spain; ^7^School of Psychological Sciences, Monash University, Melbourne, VIC, Australia

**Keywords:** cocaine dependence, motivation for treatment, personality disorders, gray matter, prefrontal cortex, striatum

## Abstract

Addiction treatment is a long-term goal and therefore prefrontal–striatal regions regulating goal-directed behavior are to be associated with individual differences on treatment motivation. We aimed at examining the association between gray matter volumes in prefrontal cortices and striatum and readiness to change at treatment onset in cocaine users with and without personality disorders. Participants included 17 cocaine users without psychiatric comorbidities, 17 cocaine users with Cluster B disorders, and 12 cocaine users with Cluster C disorders. They completed the University of Rhode Island Change Assessment Scale, which measures four stages of treatment change (precontemplation, contemplation, action, and maintenance) and overall readiness to change, and were scanned in a 3 T MRI scanner. We defined three regions of interest (ROIs): the ventromedial prefrontal cortex (including medial orbitofrontal cortex and subgenual and rostral anterior cingulate cortex), the dorsomedial prefrontal cortex (i.e., superior medial frontal cortex), and the neostriatum (caudate and putamen). We found that readiness to change correlated with different aspects of ventromedial prefrontal gray matter as a function of diagnosis. In cocaine users with Cluster C comorbidities, readiness to change positively correlated with gyrus rectus gray matter, whereas in cocaine users without comorbidities it *negatively* correlated with rostral anterior cingulate cortex gray matter. Moreover, maintenance scores positively correlated with dorsomedial prefrontal gray matter in cocaine users with Cluster C comorbidities, but *negatively* correlated with this region in cocaine users with Cluster B and cocaine users without comorbidities. Maintenance scores also negatively correlated with dorsal striatum gray matter in cocaine users with Cluster C comorbidities. We conclude that the link between prefrontal–striatal gray matter and treatment motivation is modulated by co-existence of personality disorders.

## Introduction

Cocaine addiction is associated with gray matter abnormalities in prefrontal and striatal brain regions (1), which regulate motivation toward rewards and goals (2, 3). Striatal regions encode the current value of tangible rewards, whereas prefrontal regions represent more abstract rewards and long-term goals (4). In the striatum, ventral striatal regions encode the motivational value of rewards, whereas dorsal striatal aspects are central for habit learning and habit-based control of instrumental behavior (5, 6). In the prefrontal cortex, ventromedial aspects represent the subjective value of rewards and goals and orchestrate goal-directed behavior (7–9), whereas dorsomedial aspects are essential to address conflict and to maintain motivation for challenges and goals over time (10–12). Cocaine use is generally associated with reductions in ventromedial and dorsomedial prefrontal gray matter and with enlargement of striatal gray matter (1, 13, 14). These neural adaptations would make cocaine users more sensitive to immediate rewards and habits, and less driven by long-term goals (15, 16).

Substance addiction treatment is a long-term goal, and therefore prefrontal and striatal features are to be associated with individual differences in baseline motivation for treatment (17, 18). Accordingly, there is a significant association between gray matter in prefrontal regions and self-reported readiness to change [measured with a questionnaire of the stages of self-change model; (19)] in individuals with alcohol dependence (20). However, in cocaine users the link between prefrontal–striatal systems and motivated behavior as measured by a behavioral inventory sensitive to prefrontal systems dysfunction is modulated by the co-existence of different types of personality disorders (i.e., Cluster B and Cluster C personality disorders) (21). These personality disorders are themselves associated with specific gray matter abnormalities. Cluster B (i.e., impulsive) disorders are associated with significantly reduced gray matter in orbitofrontal and dorsomedial prefrontal regions (22–24). Conversely, the personality features of Cluster C disorders (i.e., anxiousness, compulsivity) are positively associated with gray matter volumes in the orbitofrontal cortex and the striatum (25, 26). These brain structural differences are paralleled by different profiles of goal-driven behavior. Cocaine users with Cluster B comorbidities (similar to cocaine users without comorbidities) exhibit significantly poorer executive function performance relative to healthy controls, whereas cocaine users with Cluster C comorbidities are relatively more preserved (27). The latter finding is reminiscent of evidence showing that Cluster C comorbidities are associated with better addiction treatment outcomes (28). Therefore, the link between prefrontal–striatal gray matter and motivation for treatment is likely to be modulated by personality comorbidities.

This study was aimed to explore the association between readiness to change at treatment onset and gray matter volumes in ventromedial and dorsomedial prefrontal cortices and striatum among individuals with cocaine dependence with and without comorbid personality disorders. We also explored the association between dimensional scores of stages of change (precontemplation, contemplation, action, and maintenance) and gray matter in the same regions. We hypothesized that readiness to change would positively correlate with prefrontal gray matter (relevant to goal pursue), and negatively correlate with striatal gray matter (relevant to reward sensitivity and habit learning). Moreover, we hypothesized that the type of co-existing personality disorder (Cluster B versus Cluster C disorders) would modulate the association between readiness to change and prefrontal and striatal gray matter.

## Materials and Methods

### Participants

Forty-six cocaine-dependent individuals (17 without current psychiatric comorbidities, 17 with comorbid personality disorders from Cluster B, and 12 with comorbid personality disorders from Cluster C) participated in this study. Cocaine users were recruited through consecutive admissions to the clinic “Centro Provincial de Drogodependencias (CPD)” in Granada (Spain), which provides psychosocial treatment for substance use disorders in an outpatient setting. The inclusion criteria were defined as follows: (i) age range between 18 and 45 years old; (ii) IQ levels above or equal to 80 – as measured by the Kaufman Brief Intelligence Test (K-BIT) (29); (iii) meeting DSM-IV criteria for cocaine dependence – as assessed by the Structured Clinical Interview for DSM-IV Disorders Clinician Version (SCID) (30); (iv) being treatment commencers; and (v) abstinence duration >15 days. Abstinence was confirmed by twice weekly urine tests plus an *ad hoc* test on the scanning day. Inclusion of cocaine users with personality disorders was restricted to diagnoses pertaining to Cluster B and Cluster C, which are the more prevalent in this population. Comorbid Axis I disorders were assessed with the SCID. Axis II disorders were assessed using the International Personality Disorders Examination [(31); Spanish version by López-Ibor et al. (32)]. The exclusion criteria for all these groups were: (i) current Axis I disorders – with the exceptions of stimulant abuse or dependence, alcohol abuse, nicotine dependence, and attention-deficit and hyperactivity disorder (ADHD) – as measured by the Conners’ Adult ADHD Diagnostic Interview for DSM-IV [CAADID; (33)]; (ii) history of head injury or neurological, infectious, systemic, or any other diseases affecting the central nervous system; (iii) having followed other treatments within the 2 years preceding the study onset; and (iv) having entered treatment by court request.

The study was approved by the research ethical committee of the University of Granada and all the participants gave written informed consent according to the Declaration of Helsinki (34).

### Instruments

#### Patterns of drug use

Data regarding lifetime amount and duration of drug use were self-reported by participants and collected using the Interview for Research on Addictive Behavior (35). This interview provides an estimation of monthly use of each substance during regular use (e.g., grams for cocaine, standard alcohol units for alcohol, number of cigarettes for tobacco) and total duration of use of each substance (in months).

#### University of Rhode Island Change Assessment Scale

This is a 32-item self-report questionnaire that measures the current motivational state of the individual in relation to four theoretically driven stages of change relevant to drug addiction treatment: precontemplation, contemplation, action, and maintenance (19). Precontemplation reflects poor consideration of the need to change. Contemplation reflects early disposition to begin change, even in the presence of ambivalence. Action reflects current commitment with treatment-related required changes. Finally, maintenance reflects willingness to sustain treatment change-related efforts through time. The questionnaire provides dimensional estimations of each of these stages of change, and a composite measure of overall readiness to change. The readiness to change measure is calculated by subtracting precontemplation scores from the sum of contemplation, action, and maintenance subscales.

### MRI acquisition and pre-processing

Participants were scanned on a 3 T whole body MRI scanner (Phillips Achieva X-series) operating with an eight-channel-phased array head coil. For each participant, a 3D volume was acquired using a T1-weighted turbo-gradient-echo sequence (3D-TFE) in the sagittal plane, with a 0.94 mm × 0.94 mm × 1.0 mm resolution (160 slices, FOV = 240 mm × 240 mm, matrix 256 × 256), TR = 8.3 ms, TE = 3.8 ms, TI = 1022.6264 ms, and flip angle = 8°. This sequence was optimal for reducing motion sensitivity, susceptibility artifacts, and field inhomogeneities. Structural imaging data were pre-processed and analyzed using statistical parametric mapping 8 (SPM8)^1^ implemented in Matlab R2007b (MathWorks, Natick, MA, USA). We used the VBM8 toolbox^2^ to segment raw images and extract probabilistic maps of gray matter, white matter, and cerebrospinal fluid; normalize the gray matter segments using DARTEL normalization to a gray matter template in MNI space (36); modulate normalized gray matter images with the Jacobian determinants (from the flow-fields derived from the normalization step) to restore volumetric information; and finally smooth images with a 3-D Gaussian filter of 8 mm full-width at half maximum.

### Data analysis

#### Behavioral analyses

Behavioral data were analyzed with the Statistical Package for the Social Sciences version 15.0 (SPSS; Chicago, IL, USA). One-way analyses of variance (ANOVAs) were conducted to compare the three diagnostic groups on relevant demographic (i.e., age, IQ), drug use, and University of Rhode Island Change Assessment Scale (URICA) variables. Drug use analyses were restricted to those substances used by more than 10% of the participants. Finally, the association between the different subscales of the instrument and the measure of readiness to change with age, gender, comorbid ADHD, and severity of tobacco, alcohol, and cocaine consumption (defined as the standardization of the product of monthly amount and duration of use of each drug) were explored by using bivariate correlations or Mann–Whitney tests.

#### Image analyses

Our image analyses were initially performed at the whole-brain level, both assessing the correlations between our psychometric measurements and regional gray matter volumes for the whole group of participants and specifically for each subgroup. Subsequently, we restricted our analyses to three bilateral regions of interest (ROIs): the ventromedial and dorsomedial prefrontal cortices and the neostriatum. These ROIs were created using the coordinates provided by the Talairach Daemon database (37, 38) included in WFU PickAtlas Tool Version 2.5.2^3^ (39). Specifically, in agreement with previous research (40, 41), the ventromedial prefrontal ROI included the medial orbitofrontal cortex and the subgenual and rostral anterior cingulate cortices. The dorsomedial prefrontal ROI included the superior medial frontal cortex, such that there was no overlap with the ventromedial prefrontal ROI. The neostriatum ROI included caudate and putamen nuclei. Figure S1 in Supplementary Material displays the ROIs utilized in the study. A one-way ANOVA model was first conducted to examine between-group differences in the voxel-wise volumes within our ROIs. Next, we tested the association between our psychometric assessments and voxel-wise gray matter volumes within each ROI using multiple regression analyses. For each variable, we conducted four models: one using the whole sample, and other three analyzing the associations between the variables within each group. In all the analyses, severity of cocaine and alcohol use, and total gray matter volume (TGMV) were modeled as linear confounds. Significance threshold was set at *p* < 0.05 after family-wise error correction for multiple comparisons across the whole-brain (pFWE < 0.05) or the voxels of the different ROIs [i.e., using small volume correction (SVC) procedures (pFWE-SVC < 0.05)].

## Results

### Behavioral analyses

There were no significant differences between the groups in any of the variables analyzed (Tables 1 and 2). Likewise, no significant associations were found between URICA scores and potential confounders including age, gender, comorbid ADHD, and severity of tobacco, cocaine, or alcohol consumption.

**Table 1:** **Demographic and drug use characteristics of the sample**.

	CDI (*n* = 17)		CDI + Cluster B (*n* = 17)		CDI + Cluster C (*n* = 12)		*F*	*p*
	Mean	SD	Mean	SD	Mean	SD	
**DEMOGRAPHICS**								
Gender (male/female)	16/1		9/8		12/0			
ADHD	1		3		0			
Age	34.53	7.34	34.18	8.85	33.67	5.93	0.045	0.956
Total IQ	95.76	8.62	97.76	9.93	98	8.01	0.294	0.747
Verbal IQ	100.53	7.58	101.59	11.11	101.50	7.42	0.069	0.933
Performance IQ	97.59	10.02	97.88	8.27	99.83	13.13	0.186	0.831
**PATTERNS OF DRUG USE**								
Cocaine
Cocaine grams per month	17.74	25.99	21.18	27.56	8.25	7.57	1.107	0.340
Cocaine duration of use (months)	54.79	60.04	48.41	46.76	69.33	54.52	0.538	0.588
Cocaine duration of abstinence (months)	2.86	5.57	4.74	6.06	1.67	0.84	1.382	0.262
Alcohol
Alcohol standard units per month	25	29.92	25.53	40.09	30.33	38.87	0.087	0.916
Alcohol duration of use (months)	82.76	95.94	51.74	70.52	70	73.05	0.621	0.542
Tobacco
Tobacco cigarettes per month	464.71	448.07	334.65	254.04	264.17	227.69	1.369	0.265
Tobacco duration of use (months)	111.18	123.59	119.88	122.21	88.75	91.60	0.262	0.771

*CDI = cocaine dependence individuals; ADHD = attention-deficit hyperactivity disorder*.

**Table 2:** **University of Rhode Island Change Assessment Scale scores for the four individual scales and the measure of readiness to change in the three study groups**.

	CDI		CDI + Cluster B		CDI + Cluster C		*F*	*p*
	Mean	SD	Mean	SD	Mean	SD	
**VARIABLES**								
Precontemplation	1.99	0.51	1.68	0.34	1.80	0.39	2.354	0.107
Contemplation	4.30	0.46	4.42	0.51	4.28	0.53	0.353	0.705
Action	4.31	0.42	4.35	0.31	4.06	0.29	2.746	0.075
Maintenance	3.80	0.57	3.86	0.74	3.71	0.54	0.217	0.806
Readiness to change	10.41	1.42	10.96	1.35	10.24	1.02	1.262	0.293

*CDI = cocaine dependence individuals*.

### Image analyses

#### Whole-brain analyses

Whole-brain analyses did not show significant correlations with stages of change subscales scores or readiness to change at pFWE < 0.05. For illustrative purposes, the results of these analyses at a more lenient significance threshold (*p* < 0.001, uncorrected) are reported in Table S1 in Supplementary Material.

#### ROIs analyses

##### Group differences

We did not find any significant between-group differences in the volume of the ROIs assessed.

##### Association between brain volumes and motivation for treatment

The results of these analyses can be found in Table 3.

**Table 3:** **Significant correlations between the subscales of the questionnaire and readiness to change and gray matter volumes in the targeted regions of interest**.

Variable	Correlation	*x*	*y*	*z*	*k*	*t*
**PRECONTEMPLATION**						
Whole sample
Left superior medial frontal gyrus	+	−12	63	4	790	4.94
**MAINTENANCE**						
CDI without current psychiatric comorbidities
Right superior medial frontal gyrus	−	6	53	30	276	5.90
CDI with comorbid personality disorders from Cluster B
Right superior medial frontal gyrus	−	5	24	55	76	6.12
CDI with comorbid personality disorders from Cluster C
Right striatum	−	33	−7	1	105	10.05
Left superior medial frontal gyrus	+	−6	47	33	1259	11.04
**READINESS TO CHANGE**						
CDI without current psychiatric comorbidities
Right rostral anterior cingulate cortex	−	6	26	16	203	5.87
CDI with comorbid personality disorders from Cluster C
Right gyrus rectus	+	11	32	−18	174	10.53

*CDI = cocaine dependence individuals. Stereotaxic coordinates are those listed in SPM8. The corresponding anatomical names were obtained using the tool AAL in MRICron (42)*.

**Figure 1:**
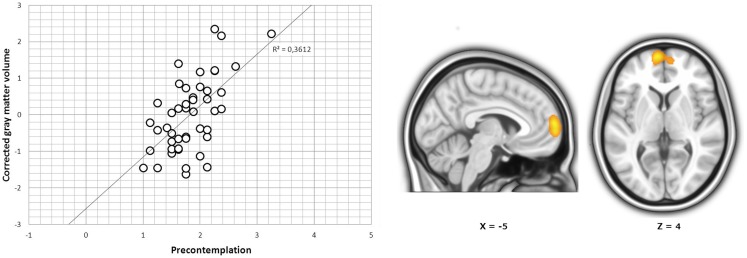
***r* Coefficients and cluster of correlation between the dorsomedial prefrontal cortex and the measure of precontemplation in the whole sample**. Results are overlaid on sagittal and axial sections of a normalized brain, and the numbers correspond to the “*x*” and “*z*” coordinates in MNI space.

**Figure 2:**
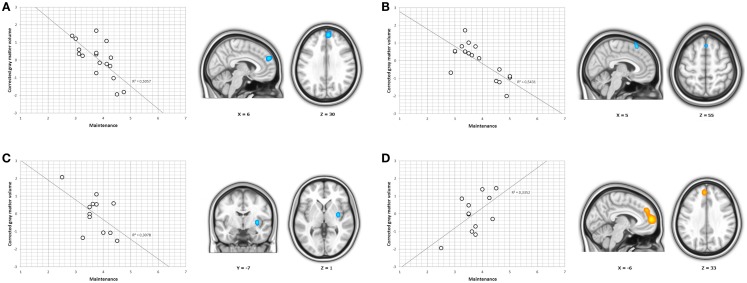
***r* Coefficients and clusters of correlation found using the measure of maintenance**. **(A)** *r* Coefficients and cluster of correlation between the dorsomedial prefrontal cortex and the measure of maintenance in cocaine-dependent individuals without current psychiatric comorbidities. **(B)** *r* Coefficients and cluster of correlation between the dorsomedial prefrontal cortex and the measure of maintenance in cocaine-dependent individuals with comorbid personality disorders from Cluster B. **(C)** *r* Coefficients and cluster of correlation between the neostriatum and the measure of maintenance in cocaine-dependent individuals with comorbid personality disorders from Cluster C. **(D)** *r* Coefficients and cluster of correlation between the dorsomedial prefrontal cortex and the measure of maintenance in cocaine-dependent individuals with comorbid personality disorders from Cluster C. Results are overlaid on sagittal, coronal, and axial sections of a normalized brain, and the numbers correspond to the “*x*,” “*y*,” and “*z*” coordinates in MNI space.

*Stages of changes subscales scores*. In the whole sample, we found a significant positive correlation between precontemplation scores and a cluster located in the left dorsomedial prefrontal cortex (peak at *x*, *y*, *z* = −12, 63, 4; *t*_41_ = 4.94; pFWE-SVC < 0.05; Figure 1). Subgroup analyses further showed that in cocaine-dependent individuals without comorbidities, there was a significant negative correlation between maintenance scores and one cluster located in the dorsomedial prefrontal cortex, specifically in the right superior medial frontal gyrus (peak at *x*, *y*, *z* = 6, 53, 30; *t*_12_ = 5.90; pFWE-SVC < 0.05; Figure 2A). In cocaine-dependent individuals with Cluster B personality disorders, we also found a significant negative correlation between maintenance scores and regional gray matter in the right superior medial frontal gyrus (peak at *x*, *y*, *z* = 5, 24, 55; *t*_12_ = 6.12; pFWE-SVC < 0.05; Figure 2B). Finally, in cocaine-dependent individuals with Cluster C personality disorders, we found a significant negative correlation between maintenance scores and a gray matter cluster located in the right striatum (in the right dorsal caudal putamen, peak at *x*, *y*, *z* = 33, −7, 1; *t*_7_ = 10.05; pFWE-SVC < 0.05; Figure 2C), and a positive correlation between maintenance scores and regional gray matter within the superior medial frontal gyrus of the left hemisphere (peak at *x*, *y*, *z* = −6, 47, 33; *t*_7_ = 11.04; pFWE-SVC < 0.05; Figure 2D). We found no significant results for precontemplation, contemplation, or action scores in subgroup analyses.

**Figure 3:**
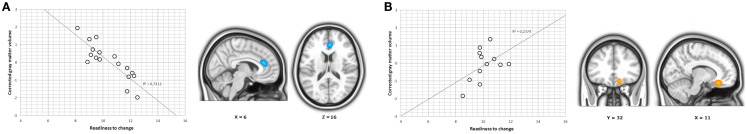
***r* Coefficients and clusters of correlation found using the measure of readiness to change**. **(A)** *r* Coefficients and cluster of correlation between the ventromedial prefrontal cortex and the measure of readiness to change in cocaine-dependent individuals without current psychiatric comorbidities. **(B)** *r* Coefficients and cluster of correlation between the ventromedial prefrontal cortex and the measure of readiness to change in cocaine-dependent individuals with comorbid personality disorders from Cluster C. Results are overlaid on sagittal, coronal, and axial sections of a normalized brain, and the numbers correspond to the “*x*,” “*y*,” and “*z*” coordinates in MNI space.

*Readiness to change*. We found no significant results in the whole sample. Subgroup analyses showed that in cocaine-dependent individuals without comorbidities, there was a significant negative correlation between readiness to change and a cluster located in the right rostral anterior cingulate cortex (peak at *x*, *y*, *z* = 6, 26, 16; *t*_12_ = 5.87; pFWE-SVC < 0.05; Figure 3A). In cocaine-dependent individuals with Cluster C personality disorders, we found a significant positive correlation between readiness to change and a gray matter cluster located in the right gyrus rectus (peak at *x*, *y*, *z* = 11, 32, −18; *t*_7_ = 10.53; pFWE-SVC < 0.05; Figure 3B).

## Discussion

We found that readiness to change at treatment onset correlates with gray matter volume in the ventromedial prefrontal cortex in different ways for different diagnostic groups. In cocaine users with Cluster C comorbidities, readiness to change positively correlated with gray matter volume in the gyrus rectus, whereas in cocaine users without comorbidities readiness to change *negatively* correlated with gray matter volume in the rostral anterior cingulate cortex. Moreover, scores of maintenance correlate with gray matter volume in the dorsomedial prefrontal cortex as well in different ways for different diagnostic groups. Maintenance scores *negatively* correlated with gray matter in the right superior medial frontal gyrus in cocaine users without comorbidities and cocaine users with Cluster B comorbidities, whereas they positively correlated with gray matter in the left superior medial frontal gyrus in cocaine users with Cluster C comorbidities. Further, maintenance scores negatively correlated with dorsal striatum gray matter in cocaine users with Cluster C comorbidities. These findings indicate that the link between readiness to change at treatment onset and prefrontal–striatal brain structure is manifested in ventromedial prefrontal but not dorsomedial prefrontal regions. However, the dorsomedial prefrontal region is linked to specific stages of change, particularly to maintenance. Moreover, the direction of the link between treatment motivation and prefrontal and striatal gray matter is modulated by the presence and the type of comorbidities with personality disorders.

In cocaine users with Cluster C comorbidities the association between prefrontal and striatal brain structure and motivation for treatment was in the expected direction. Greater ventromedial prefrontal gray matter was associated with more readiness to change, and greater dorsomedial prefrontal gray matter was associated with more maintenance or willingness to make sustained treatment-related efforts. Greater gray matter in the dorsal striatum was nonetheless associated with poorer maintenance scores. These findings are in fitting with our original assumptions and with the function of these regions as revealed by human lesion studies. Ventromedial prefrontal lesions (encompassing the gyrus rectus region) associate with insensitivity to long-term outcomes (43, 44). Conversely, superior frontal lesions associate with apathy symptoms (45). Moreover, greater striatal volumes associate with increased habit learning and decreased goal-related learning in healthy individuals (46). Functional imaging studies have as well shown that the ventromedial prefrontal cortex is central for orchestrating goal-directed behavior, whereas the dorsal striatum is central for habit-based control of behavior (7–9). Therefore, in cocaine users with Cluster C disorders lower ventromedial prefrontal gray matter may signal less ability to align behavior with long-term goals, whereas greater striatal gray matter may signal lingering sensitivity to habitual behaviors (which are incompatible with treatment goals). In direct contrast with the above findings, ventromedial prefrontal gray matter specifically in the right rostral anterior cingulate cortex negatively correlated with readiness to change in cocaine users without comorbidities. The direction of this correlation was not expected and therefore future studies are warranted to seek replication. However, lower gray matter in this region has been previously associated with impaired insight (i.e., low emotional awareness) in cocaine-dependent users (47). Therefore, greater gray matter levels in the right rostral anterior cingulate may purportedly facilitate emotional awareness of the difficulty of treatment, and therefore hinder motivation for treatment (48).

In both cocaine users without comorbidities and cocaine users with Cluster B comorbidities, we found that greater dorsomedial prefrontal gray matter (specifically in the right superior medial frontal gyrus) was linked to *lower* scores of maintenance, indicating lower willingness to sustain therapeutic efforts (19). The (unpredicted) direction of this correlation suggests that in these populations greater dorsomedial prefrontal gray matter is associated with treatment-wise disadvantageous features. These results are reminiscent of those of a previous study in which cocaine users with greater white matter in the right inferior frontal gyrus also showed less motivation for treatment (49). In previous work, we have as well shown that right dorsomedial prefrontal cortex (BA 8) gray matter volumes are positively associated with higher levels of negative urgency (i.e., negative emotion driven impulsivity), in direct contrast with the negative associations found in healthy controls (1). Moreover, gray matter in right dorsomedial prefrontal regions is also positively correlated with antisocial traits (50, 51). Therefore, it is reasonable to speculate that the link between greater dorsomedial prefrontal volumes and lower motivation for change in cocaine users without comorbidities and those with Cluster B comorbidities is mediated by the association between this region and trait characteristics known to detrimentally impact treatment outcome.

We did not find major results in the whole sample, with the exception of a positive correlation between left dorsomedial prefrontal gray matter and precontemplation scores. Due to the role of this region in conflict processing, this correlation is likely reflective of conflict assessment, which is central for behavioral change (10). However, most of our findings point to specific associations between frontal–striatal gray matter and treatment motivation in each of the diagnostic subpopulations. To the extent that gray matter represents the status of motivational dispositions and cognitive and emotional features, these findings point to the need of matching diagnostic subpopulations to specific treatment options. For example, in cocaine users with Cluster C disorders stimulation of ventromedial and dorsomedial prefrontal regions will be purportedly linked to increased treatment motivation and relaxation of habits. However, in Cluster B and non-comorbid cocaine users, inhibition of rostral anterior cingulate and dorsomedial prefrontal regions will be purportedly linked to greater commitment with treatment via reduction of urgency or negatively laden insights. These notions may nurture the design of future studies aimed at applying cortical stimulation or inhibition techniques (i.e., direct current or transcranial magnetic stimulations) or neurofeedback to modulate treatment motivation in stimulant addiction. They could be as well useful to match cognitive interventions to diagnostic subpopulations. For example, episodic future thinking training has shown stimulatory effects in the ventromedial prefrontal regions that positively correlate with readiness to change and maintenance in cocaine users with Cluster C comorbidities (52). Moreover, goal-directed implicit learning training decreases activation in the medial frontal regions negatively correlated with maintenance in cocaine users without comorbidities and with Cluster B comorbidities (53).

This study is the first to demonstrate an association between prefrontal–striatal gray matter and readiness to change in the context of cocaine addiction. The main findings are that this association is primarily observed in ventromedial prefrontal regions, and that the direction of the link is modulated by the co-existence of personality disorders and the type of disorder. Strengths include a comprehensive phenotypic characterization and adequate control of confounding variables through eligibility criteria (i.e., acute use, other substance dependences, or Axis I comorbidities) or statistical control (i.e., covariation of severity of use of cocaine and alcohol). The main limitation is the relatively small sample size, which may have precluded us from finding significant effects in whole-brain analyses. Another relevant limitation is the homogeneity of the sample in relation to treatment status, since all participants were treatment seekers and treatment commencers. These features may have produced a ceiling effect on treatment motivation scores, which precluded us from finding group differences on behavioral scales and relatively small effects in correlations between behavior and gray matter volumes. Future studies with larger and more heterogeneous treatment samples are therefore required to replicate these results and to clarify whether the negative association between greater dorsomedial prefrontal brain volumes and motivation for treatment are mediated by common trait features of cocaine addiction (but atypical of Cluster C disorders) like impulsivity or emotional lability.

## Conflict of Interest Statement

The authors declare that the research was conducted in the absence of any commercial or financial relationships that could be construed as a potential conflict of interest.

## Supplementary Material

The Supplementary Material for this article can be found online at http://www.frontiersin.org/Journal/10.3389/fpsyt.2014.00052/abstract

Table S1 **Significant correlations between the subscales of the questionnaire and the measure of readiness to change and gray matter volumes at *p* < 0.001 (uncorrected)**.

Figure S1 **Regions of interest masks used in the analyses for the ventromedial prefrontal cortex (green), the dorsomedial prefrontal cortex (blue), and the neostriatum (yellow)**. Masks are overlaid on sagittal and coronal sections of a normalized brain, and the numbers correspond to the “*x*” and “*y*” coordinates in MNI space.

## Footnotes

^1^http://www.fil.ion.ucl.ac.uk/spm/software/spm8/

^2^http://dbm.neuro.uni-jena.de/vbm/

^3^http://www.ansir.wfubmc.edu
