# Supplementary material for: Prefrontal Gray Matter and Motivation for Treatment in Cocaine-Dependent Individuals with and without Personality Disorders
Source: Front Psychiatry. 2014 May 20;5:52. doi: 10.3389/fpsyt.2014.00052 (PMC4032993; doi:10.3389/fpsyt.2014.00052)
Supplement: Table S1 — Significant correlations between the subscales of the questionnaire and the measure of readiness to change and gray matter volumes at p < 0.001 (uncorrected). [file DataSheet1.DOCX]

**Supplementary material**

**Table 1.** Significant correlations between the subscales of the questionnaire and the measure of readiness to change and gray matter volumes at p<0.001 (uncorrected).

|  | **Correlation** | **x** | **y** | **z** | **k** | **t** |
| --- | --- | --- | --- | --- | --- | --- |
| Precontemplation | | | | | | |
| CDI without current psychiatric comorbidities | | | | | | |
| Right inferior temporal cortex | + | 47 | -73 | -3 | 38 | 4.91 |
| Left middle frontal gyrus | - | -26 | 32 | 49 | 18 | 5.83 |
| CDI with comorbid personality disorders from Cluster B | | | | | | |
| Right cerebelum | + | 21 | -64 | -45 | 124 | 4.66 |
| Right superior parietal cortex | - | 23 | -66 | 52 | 63 | 7.01 |

| CDI with comorbid personality disorders from Cluster C |
| --- |

| Left inferior frontal operculum | + | -60 | 12 | 9 | 27 | 10.69 |
| --- | --- | --- | --- | --- | --- | --- |
| Left inferior frontal triangularis | - | -54 | -31 | 9 | 28 | 12.41 |

| Contemplation |
| --- |
| CDI without current psychiatric comorbidities |

| Right superior occipital cortex | + | 21 | -61 | 49 | 84 | 5.09 |
| --- | --- | --- | --- | --- | --- | --- |
| Right postcentral gyrus | - | 48 | -7 | 34 | 435 | 6.47 |

| CDI with comorbid personality disorders from Cluster B |
| --- |

| Right superior occipital cortex | + | 33 | -75 | 48 | 7 | 5.40 |
| --- | --- | --- | --- | --- | --- | --- |
| Right precentral gyrus | - | 38 | -9 | 54 | 18 | 5.00 |

| CDI with comorbid personality disorders from Cluster C |
| --- |

| Right superior temporal cortex | + | 54 | -15 | 3 | 138 | 10.86 |
| --- | --- | --- | --- | --- | --- | --- |
| Left angular gyrus | - | -62 | -54 | 34 | 554 | 13.41 |

| Action |
| --- |
| CDI without current psychiatric comorbidities |

| Right middle frontal gyrus | + | 33 | 26 | 39 | 36 | 6.11 |
| --- | --- | --- | --- | --- | --- | --- |
| Left postcentral gyrus | - | -60 | -9 | 34 | 201 | 7.35 |

| CDI with comorbid personality disorders from Cluster B |
| --- |

| Right cerebelum | + | 36 | -45 | -42 | 185 | 7.66 |
| --- | --- | --- | --- | --- | --- | --- |
| Right middle temporal cortex | - | 65 | -54 | 15 | 17 | 5.53 |

| CDI with comorbid personality disorders from Cluster C |
| --- |

| Right superior parietal cortex | + | 15 | -60 | 62 | 25 | 9.42 |
| --- | --- | --- | --- | --- | --- | --- |
| Left calcarine | - | -17 | -102 | -2 | 59 | 6.86 |

| Maintenance |
| --- |
| CDI without current psychiatric comorbidities |

| Right fusiform gyrus | + | 33 | -1 | -39 | 34 | 6.63 |
| --- | --- | --- | --- | --- | --- | --- |
| Right superior medial frontal gyrus | - | 6 | 53 | 30 | 85 | 5.90 |

| CDI with comorbid personality disorders from Cluster B |
| --- |

| Left middle frontal gyrus | + | -24 | 3 | 61 | 13 | 5.23 |
| --- | --- | --- | --- | --- | --- | --- |
| Right supplementary motor area | - | 5 | 24 | 55 | 55 | 6.12 |

| CDI with comorbid personality disorders from Cluster C |
| --- |

| Left superior medial frontal gyrus | + | -6 | 47 | 33 | 71 | 11.04 |
| --- | --- | --- | --- | --- | --- | --- |
| Left supramarginal cortex | - | -54 | -43 | 36 | 71 | 10.55 |

|  | **Correlation** | **x** | **y** | **z** | **k** | **t** |
| --- | --- | --- | --- | --- | --- | --- |
| Readiness to change | | | | | | |
| CDI without current psychiatric comorbidities | | | | | | |
| Right parahippocampal gyrus | + | 33 | -16 | -23 | 76 | 5.12 |
| Right postcentral gyrus | - | 47 | -7 | 34 | 444 | 7.41 |
| CDI with comorbid personality disorders from Cluster B | | | | | | |
| Left precuneus | + | -6 | -46 | 43 | 162 | 8.70 |
| Left middle temporal cortex | - | -62 | 2 | -24 | 16 | 5.27 |
| CDI with comorbid personality disorders from Cluster C | | | | | | |
| Right gyrus rectus | + | 11 | 32 | -18 | 89 | 10.53 |
| Left supramarginal cortex | - | -56 | -48 | 36 | 514 | 15.87 |

CDI=Cocaine Dependence Individuals. Stereotaxic coordinates are those listed in SPM8. The corresponding anatomical names were obtained using the tool aal in MRICron (Rorden and Brett, 2000).
