# Supplementary figures and images for: Prefrontal Gray Matter and Motivation for Treatment in Cocaine-Dependent Individuals with and without Personality Disorders
Source: Front Psychiatry. 2014 May 20;5:52. doi: 10.3389/fpsyt.2014.00052 (PMC4032993; doi:10.3389/fpsyt.2014.00052)

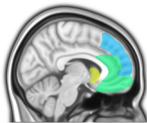

**X = 8**

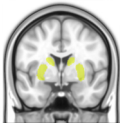

**Y = 0**

Supplement: Figure S1 — Regions of interest masks used in the analyses for the ventromedial prefrontal cortex (green), the dorsomedial prefrontal cortex (blue), and the neostriatum (yellow). Masks are overlaid on sagittal and coronal sections of a normalized brain, and the numbers correspond to the “x” and “y” coordinates in MNI space. [file Presentation1.PDF]
